# Carbon and nitrogen isotopic composition of commercial dog food in Brazil

**DOI:** 10.7717/peerj.5828

**Published:** 2019-02-20

**Authors:** Leonardo de Aro Galera, Adibe Luiz Abdalla Filho, Luiza Santos Reis, Janaina Leite de Souza, Yeleine Almoza Hernandez, Luiz Antonio Martinelli

**Affiliations:** Center for Nuclear Energy in Agriculture, University of São Paulo, Piracicaba, São Paulo, Brazil

**Keywords:** Carbon-13, Nitrogen-15, Food composition, Pets, Labels, Pet food market, Corn, Chicken, Bovine, Grains

## Abstract

**Background:**

Brazil is a low- to medium-income country and has the second largest pet food market in the world with 8% of world pet food consumption. The lowest-income social class spends around 17% of their domestic budget on pet food and other items related to pets. Consumers are frequently misled by advertising as there is no precise information about the main sources of protein, carbohydrates and fat in the labels, and the Brazilian pet food industry can legally claim that their products contain certain items like salmon or beef even if they use just a flavoring compound.

**Methods:**

The stable isotope methodology compares the stable isotope ratios of carbon (^13^C/^12^C) and nitrogen (^15^N/^14^N) between source and product. The ^13^C/^12^C ratio of a specific product (e.g., dog food) reveals the proportions of C_4_ (maize) and C_3_ (soybean, rice and wheat) plants in that product and the ^15^N/^14^N ratio reveals the proportion of the compounds derived from animals. With this isotopic data, we used MixSIAR, a Bayesian stable isotope-mixing model, to estimate the proportion of maize, grains, poultry and beef in dog food.

**Results:**

The δ^13^C values of dry dog food ranged from −24.2‰ to −12.8‰, with an average (± standard-deviation) of −17.1‰ ± 2.8‰. The δ^13^C values of wet pet food ranged from −25.4‰ to −16.9‰, with an average (± standard-deviation) of −21.2‰ ± 2.4‰, which was significantly lower (*p* < 0.01). The δ^15^N values of the dry and wet food ranged from 1.7‰ to 4.2‰, and from 0.5‰ to 5.5‰, respectively. The average δ^15^N values of dry food (2.9‰ ± 0.5‰) was not higher than the wet food (2.6‰ ± 1.3‰) (*p* > 0.01). The output of the MixSIAR showed a low proportion of bovine products in dry dog food samples. On the other hand, poultry was obviously the dominant ingredient present in most of the samples. Maize was the second dominant ingredient. Wet and dry dog food showed similar isotopic analysis results. The only difference was a lower proportion of maize and higher proportion of grains in wet dog food.

**Discussion:**

The main finding is that dog food in Brazil is mostly made of approximately 60% (ranging from 32% to 86%) animal-based and 40% (ranging from 14% to 67%) plant-based products. Poultry and maize are the main ingredients. Poultry is added as a by-product or meal, which avoids competition between dogs and humans for meat products, while they can compete for maize. On the other hand, a large proportion of plant-based products in dog food decreases the energy and environmental footprint, since plant-based food products tend to be less harmful compared to animal-based products. Labels can mislead consumers by showing pictures of items that are not necessarily part of the product composition and by not showing the detailed information on the proportion of each ingredient. This information would allow customers to make their own choices considering their pet’s nutrition, the competition between animals and humans for resources and environmental sustainability.

## Introduction

Domestic dogs (*Canis familiaris*) and humans have shared a history of co-existence for more than 10,000 years (Freedman et al., 2014; Arendt et al., 2016; Heberlein et al., 2016). In South America, it seems that domestic dogs were introduced between 7,500 and 4,500 years BP (Prates, Prevosti & Berón, 2010); and in Brazil, the oldest known evidence of domestic dogs dated by radiocarbon is between 1,700 and 1,500 years BP (Guedes Milheira et al., 2016).

The human–dog relationship began while humans lived as nomadic hunter-gatherers. Encampments likely attracted carnivorous gray wolves (*C. lupus*)—the direct ancestors of dogs—to scavenge carcasses or track down escaped wounded animals (Freedman, Lohmueller & Wayne, 2016). These observations show that there has been a relationship between diets of humans and dogs throughout history (Rick et al., 2011; Guiry, 2012; Losey et al., 2013; McManus-Fry et al., 2016).

Nowadays, dogs are considered part of the family and thus people are spending more money to ensure good nutrition for their pets. In developed countries, more than 90% of the calories consumed by pets comes from industrialized pet food (Zicker, 2008). In developing countries, half of the pets still eat human food and table scraps, but the trend to humanize pets and strong marketing pressure by pet food companies are making this habit less common (Phillips-Donaldson, 2011). A report about pet food from Euromonitor International has shown that the pet food market is growing rapidly in developing countries (Bradley & King, 2012). According to this report, Brazil’s pet food market is now the second largest in the world with 8% of world pet food consumption, having approximately 36 million dogs corresponding to 10% of the world canine population. This fast-growing market is equal to 0.34% of the Brazilian gross domestic product, larger than the sectors for refrigerators and freezers, electronic devices and beauty products (Brazilian Association of the Pet Products Industry (ABINPET), 2015).

It seems counterintuitive that a low to medium-income country like Brazil has such a large and profitable pet market. Brazilians spend a significant part of their income on pet food and other items related to pets. People with the lowest income spend around 17% of their home budgets for pets (Brazilian Association of the Pet Products Industry (ABINPET), 2015).

The three main components of dog food are protein, carbohydrates, and fat. Protein sources can be either derived from animals or plants (Thompson, 2008). Poultry and beef are the major protein sources with animal origin, which are added to the dog food as by-products (organs not eaten by humans) or meals (ground tissues) (see Thompson, 2008 for a detailed definition). Maize, soybean, rice, and wheat are the most common sources with plant origin, and they are added in the form of flour, meal, grits, gluten, and oils. These plant species are used as sources of carbohydrates, protein, and fibers (Thompson, 2008).

However, there is a lack of information about the main sources of protein, carbohydrates, and fat provided on dog food labels. Such information is necessary in order to keep the pet food industry sustainable (Swanson et al., 2013; Okin, 2017). Producers can legally claim that their products contain certain items like salmon or beef, even if they just use a flavoring compound. When such “claims” are accompanied by graphic representations on the labels like a picture of diced beef, a “merely illustrative image” should be mentioned (Brazilian Association of the Pet Products Industry (ABINPET), 2017). Besides this, pet food labels usually list more than 40 items without mentioning the quantity of them (Thompson, 2008). Most of the labels mention that the ingredients are listed in descending order according to their importance and quantity (Brazilian Association of the Pet Products Industry (ABINPET), 2015), but not specifying the exact amounts. Therefore, consumers can be misled by these labels and advertising, buying something they did not really want.

The stable isotope methodology is based on comparisons of the stable isotope ratios of carbon (^13^C/^12^C) and nitrogen (^15^N/^14^N) in source and product considering any isotopic discrimination that may occur throughout the process (Ehleringer et al., 2015). Common uses of this methodology include studies related to animal prey-consumer (Newsome et al., 2007; Caut, Angulo & Courchamp, 2009), nutrition of modern (Nardoto et al., 2006; Valenzuela et al., 2012) and ancient hominins (Sponheimer et al., 2013), and the authentication or adulteration of food and beverage products (Rossmann, 2001).

Maize and other tropical grasses like sugarcane have a C_4_ photosynthetic pathway; while, soybean, rice, and wheat have a C_3_ photosynthetic pathway. The biochemical reactions involved in these two pathways lead to distinct ^13^C/^12^C ratios in each of these two groups of plants (Farquhar, Ehleringer & Hubick, 1989). Therefore, the ^13^C/^12^C ratio of a consumer (e.g., pets) or a product (e.g., dog food) reveals the proportion of C_4_ (maize) and C_3_ (soybean, rice, and wheat) plants in the consumer diet or in the product (DeNiro, Schoeninger & Hastorf, 1985).

Most of the territory of Brazil is within the Equator and the Tropic of Capricorn, with abundant sunlight and warm temperatures. This climatic condition favors the growth of C_4_ plants. Consequently, Brazil is a major producer of three major commercial C_4_ plants: maize, sugarcane, and forage grasses (Martinelli et al., 2010). Due to this abundance, and relatively cheap prices, C_4_ plants, like maize and sugarcane, are commonly found in all kinds of Brazilian food products, such as beer (Mardegan et al., 2013), honey (Ehleringer et al., 2015), wine (Martinelli et al., 2003), and soy sauce (Morais et al., 2018). Maize is also a main component in the diet of poultry and pork (Coletta et al., 2012), and C_4_ grasses, especially of the genus *Brachiaria* are extensively used as forages for feeding cattle (Gracindo et al., 2014; Montalvão Lima et al., 2018). Accordingly, in Brazil, higher ^13^C/^12^C ratios in food products indicate the presence of C_4_ plants like maize, grass forage, and sugarcane.

The ratio of nitrogen stable isotopes (^15^N/^14^N) increases throughout the food chain due to the preferential loss of ^14^N, thus, carnivores and products rich in protein with animal origin have higher ^15^N/^14^N ratio compared to herbivores and products containing a high proportion of plant materials (DeNiro & Epstein, 1981). Therefore, dog food containing a higher proportion of animal-based products would have a higher ^15^N/^14^N ratio related to the food rich in plant-based products.

Based on the above discussion, the main objective of this study was to use carbon and nitrogen isotopic ratios to find the origin of proteins, carbohydrates, and fats in dog food produced in the second largest pet food market in the world (Brazil).

## Material and Methods

We analyzed 61 samples of dry and 21 samples of wet dog food. All samples were bought from pet shops in Piracicaba, in the State of São Paulo, Brazil. The samples were produced by 17 and 8 dry and wet food producers, respectively. Each of these companies has several different lines of food for animals with different weights and breeds and hence they produce hundreds of types of pet food. As an example, a single multinational company produces approximately 70 different types of dog food available in the Brazilian market. Therefore, in this study, limited sub-samples of the dog food in the market were investigated. However, we tried to include food samples with different characteristics according to the ingredients described in the label, since it is mentioned that the ingredients are listed according to their importance and quantity in descending order (Brazilian Association of the Pet Products Industry (ABINPET), 2015). The name of the companies were not mentioned since our main interest was to measure the relative proportion of the main ingredients in dog foods in the Brazilian market in general.

In the laboratory, dry food samples were ground, sieved (0.15 mm), oven dried at 60 °C for 2 days, weighed and wrapped in tin capsules, while wet food samples were directly weighed and wrapped in tin capsules. The total C and N and their isotopic composition were determined at the Laboratory of Isotopic Ecology (LEI-CENA/USP) through combustion under continuous flow of helium in an elemental analyzer (model CHN1110; Carlo Erba, Milan, Italy) coupled with a mass spectrometer (model Delta Plus; Thermo Scientific, Bremen, Germany). The isotopic ratios were expressed by delta notation (δ) in parts per mil (‰), using international standards NBS19 and NBS22 for δ^13^C, and IAEA-N1 and IAEA-N2 for δ^15^N, which are based on the Vienna PeeDee Belemite limestone (for C) and atmospheric air (for N). The measurement uncertainty was estimated by the standard deviation of the internal standards of the laboratory and was of 0.09‰ for δ^13^C and 0.16‰ for δ^15^N. The isotopic ratios were calculated using the equation:

(1) $${\rm{\delta }}\,{\rm{X = }}\left[ {\left({{{{{\rm{R}}_{{\rm{sample}}}}} \over {{{\rm{R}}_{{\rm{standard}}}}}}} \right)-1} \right]\times1000$$

where “X” refers to ^13^C or ^15^N and “R_sample_” and “R_standard_” are the ^13^C/^12^C or ^15^N/^14^N ratios of sample and standard, respectively. We are aware that we may have a sub-estimation of the δ^13^C values in our samples as we haven’t extracted lipids, which are depleted in ^13^C. We believe that the ether extract of the dog food samples isn’t large enough to have a significant impact on the factors measured and MixSIAR analysis, as it comprises small amounts of the total masses (Tables 1 and 2). Post et al. (2007) reports that commonly samples with less than 15% don’t show differences in δ^13^C values before and after lipid extraction.

**Table 1:** Stable isotopic composition (expressed as ‰), C:N ratio, ether extract (%), and the median proportion of four main ingredients (poultry, beef, maize, and C_3_ grains) estimated by the MixSIAR of the dry dog foods produced by several companies.

id	δ^13^C	δ^15^N	C:N	EE	Poultry	Beef	Maize	C_3_ Grains
1	−24.2	2.6	10.6	14	0.41	0.01	0.09	0.48
2	−23.7	3.3	8.8	16	0.32	0.01	0.05	0.60
3	−23.1	3.4	9.6	16	0.39	0.01	0.05	0.54
4	−22.3	2.1	11.0	10	0.44	0.01	0.06	0.48
5	−21.6	2.3	14.9	–	0.41	0.01	0.11	0.46
6	−21.4	3.0	11.4	14	0.55	0.01	0.06	0.37
7	−21.3	2.8	7.9	18	0.61	0.01	0.05	0.33
8	−21.0	2.9	7.5	18	0.65	0.01	0.04	0.30
9	−21.0	2.3	9.2	18	0.61	0.01	0.06	0.33
10	−20.9	3.0	13.5	6	0.50	0.01	0.11	0.37
11	−20.1	3.3	8.2	18	0.73	0.01	0.05	0.21
12	−18.7	2.3	9.2	13	0.66	0.01	0.16	0.17
13	−18.7	2.3	9.2	13	0.66	0.01	0.16	0.17
14	−18.2	2.8	8.7	13	0.72	0.01	0.15	0.11
15	−18.2	2.8	8.7	14	0.72	0.01	0.16	0.11
16	−17.9	2.9	11.1	9	0.58	0.01	0.25	0.15
17	−17.9	2.9	11.1	9	0.58	0.01	0.25	0.15
18	−17.9	2.9	11.1	9	0.58	0.01	0.25	0.15
19	−17.9	2.9	11.1	9	0.58	0.01	0.25	0.15
20	−17.8	2.6	8.0	18	0.73	0.01	0.17	0.09
21	−17.8	2.6	8.5	–	0.74	0.01	0.22	0.03
22	−17.8	3.0	8.5	18	0.80	0.00	0.16	0.03
23	−17.8	2.0	9.8	14	0.74	0.00	0.21	0.04
24	−17.8	2.3	12.2	12	0.63	0.01	0.26	0.10
25	−17.8	2.5	11.0	18	0.70	0.01	0.23	0.07
26	−17.7	2.6	9.5	14	0.76	0.00	0.20	0.03
27	−17.4	3.7	12.1	9	0.65	0.01	0.27	0.07
28	−17.4	2.8	9.2	8	0.75	0.01	0.21	0.03
29	−17.2	2.3	10.0	18	0.71	0.00	0.25	0.03
30	−17.1	3.9	10.8	9	0.69	0.01	0.25	0.04
31	−17.0	3.0	11.2	7	0.67	0.01	0.28	0.04
32	−16.8	3.6	11.8	9	0.65	0.01	0.30	0.04
33	−16.8	2.1	9.4	16	0.69	0.00	0.28	0.02
34	−16.8	3.4	11.4	9	0.66	0.01	0.30	0.03
35	−16.6	2.7	11.0	18	0.65	0.01	0.31	0.03
36	−16.2	3.3	9.7	18	0.64	0.01	0.32	0.02
37	−16.2	3.3	9.7	18	0.64	0.01	0.32	0.02
38	−16.1	2.1	10.0	16	0.60	0.01	0.36	0.02
39	−15.9	2.5	8.4	18	0.60	0.01	0.36	0.02
40	−15.9	2.5	8.4	18	0.60	0.01	0.36	0.02
41	−15.9	2.5	8.4	18	0.54	0.09	0.37	0.00
42	−15.9	3.4	9.6	17	0.60	0.07	0.32	0.00
43	−15.6	3.4	9.2	17	0.57	0.08	0.33	0.00
44	−15.3	3.5	9.7	17	0.53	0.10	0.36	0.00
45	−15.2	2.7	10.2	9	0.51	0.05	0.42	0.00
46	−15.0	1.9	11.0	16	0.47	0.03	0.48	0.00
47	−14.7	2.7	9.4	18	0.45	0.07	0.46	0.00
48	−14.6	3.2	10.1	8	0.44	0.10	0.45	0.00
49	−14.6	3.2	10.1	8	0.44	0.11	0.44	0.00
50	−14.2	1.7	11.0	16	0.37	0.04	0.59	0.00
51	−14.1	3.0	9.8	14	0.38	0.11	0.50	0.00
52	−14.1	3.0	9.8	14	0.38	0.11	0.50	0.00
53	−13.6	3.3	12.7	6	0.30	0.12	0.57	0.00
54	−13.5	2.8	8.7	8	0.30	0.12	0.57	0.00
55	−13.5	3.4	10.1	8	0.30	0.19	0.50	0.00
56	−13.5	3.4	10.1	8	0.30	0.19	0.50	0.00
57	−13.5	3.4	10.1	8	0.30	0.19	0.50	0.00
58	−13.5	3.4	10.1	8	0.30	0.19	0.50	0.00
59	−13.5	3.4	10.1	8	0.30	0.19	0.50	0.00
60	−13.4	4.2	11.4	–	0.27	0.27	0.45	0.01
61	−12.8	2.8	10.8	12	0.20	0.12	0.67	0.00

**Table 2:** Stable isotopic composition (expressed as ‰), C:N ratio, ether extract (%), and the median proportion of four main ingredients (poultry, beef, maize, and C_3_ grains) estimated by the MixSIAR of the wet dog foods produced by several companies.

id	δ^13^C	δ^15^N	C:N	EE	Poultry	Beef	Maize	C_3_ Grains
1	−25.4	3.0	8.84	2	0.33	0.08	0.01	0.57
2	−23.1	2.6	5.89	–	0.35	0.05	0.01	0.57
3	−23.0	3.2	4.07	–	0.36	0.06	0.01	0.57
4	−23.0	2.9	6.07	–	0.36	0.06	0.01	0.56
5	−22.5	0.5	4.80	3	0.37	0.06	0.01	0.54
6	−22.5	3.2	9.55	3	0.40	0.06	0.01	0.51
7	−21.8	5.5	9.14	3	0.44	0.08	0.01	0.46
8	−21.2	1.0	5.27	3	0.50	0.07	0.01	0.40
9	−20.9	2.3	4.88	12	0.60	0.06	0.01	0.33
10	−20.7	2.8	5.77	3	0.65	0.05	0.01	0.29
11	−20.1	2.1	4.69	12	0.68	0.06	0.01	0.25
12	−20.1	1.3	5.12	3	0.59	0.09	0.01	0.30
13	−20.1	1.8	4.50	12	0.64	0.07	0.01	0.27
14	−19.7	3.3	5.54	3	0.77	0.05	0.01	0.17
15	−19.6	2.4	4.37	3	0.75	0.05	0.01	0.19
16	−19.2	0.7	4.66	6	0.40	0.21	0.01	0.35
17	−19.1	2.8	4.98	3	0.80	0.06	0.01	0.13
18	−18.6	2.5	6.71	6	0.69	0.12	0.01	0.16
19	−18.6	0.6	4.20	6	0.29	0.30	0.01	0.36
20	−17.8	5.2	6.79	6	0.45	0.31	0.01	0.21
21	−17.4	2.8	5.76	6	0.57	0.25	0.01	0.15

MixSIAR is a Bayesian stable isotope mixing model that uses biological tracers to estimate the proportions of determined ingredients (sources) in products (mixtures) (Ward, Semmens & Schindler, 2010). The following tracers were used in this study: ^13^C/^12^C and ^15^N/^14^N ratios (expressed as δ^13^C and δ^15^N, respectively), and the ratio C:N as mass basis. We assumed that the fractionation between the ingredients and the food is equal to 0‰; in other words, we assumed that the transformation of raw ingredients into dog food did not cause either any significant isotopic discrimination or any significant change in the C:N ratio. This assumption is based on previous studies showing that different cooking methods did not influence stable isotopes or the C:N ratio (DeNiro, Schoeninger & Hastorf, 1985; Bostic et al., 2015; Zhou et al., 2015; Royer et al., 2017).

Generally, there are dozens of ingredients listed on dog food labels. Running MixSIAR with many ingredients would not give us precise results. Therefore, the ingredients were grouped according to their similar isotopic compositions and C:N ratios as recommended by Parnell et al. (2012). The ingredients were firstly grouped based on their plant or animal origins. This grouping system is useful as each group has its distinct C:N ratio. Plants have higher C:N ratios and often lower δ^15^N values compared with animals. We further split the plant group into two sub-groups according to the photosynthetic pathway: C_3_ or C_4_, mainly because of the large difference in δ^13^C values between these two groups. One group consisted of all types of cereals except maize (C_4_), all pulses, vegetables and fruits. The other group encompassed commercial C_4_ plants, mainly maize.

The ingredients with animal origin were divided into two broad categories: beef and poultry, which include by-products, meals, and pure fat for both categories plus eggs for poultry. Pork was not included as it is rarely listed on dog food labels. Overall, four main sources were considered: C_3_ plants (grains), C_4_ plants (maize), beef, and poultry. The isotopic composition and C:N ratio were calculated based on the average values of 28 wheat, rice and soybean samples and 11 sweet maize samples for C_3_ and C_4_ plants, respectively (Supplemental Material). For bovine products, the average values of several cuts of meat in a total of 10 samples from Nardoto et al. (2011) were used. Finally, for poultry meat and products, the average values of 40 samples including offal and meat from Coletta et al. (2012) were considered. The average δ^13^C values and their standard deviations were of −27.4‰ ± 1.2‰, −11.4‰ ± 0.2‰, −10.9‰ ± 1.4‰, and −18.7‰ ± 0.6‰; the average δ^15^N values and their standard deviations were of 4.6‰ ± 2.9‰, 2.1‰ ± 0.8‰, 6.5‰ ± 1.2‰, and 3.6‰ ± 0.6‰; the average C:N values and their standard deviations were of 21.4 ± 8.6, 22.9 ± 5.6, 3.9 ± 1.2, and 4.6 ± 1.3; for C_3_ plants (grains), C_4_ plants (maize), beef, and poultry, respectively.

The Markov Chain Monte Carlo in the MixSIAR was set as follows: chain length: 3,000,000; burn-in: 1,500,000; thin: 5,000, and number of chains: 3. The error structure was set as “process” because there were no replicates among dog food samples (*N* = 1).

With these settings, the Gelman–Rubin diagnostic, that evaluates the convergence of multiple Markov chains, was <1.05 for all cases, which is the strictest condition (Gelman & Rubin, 1992); and the Geweke diagnostic, that also assesses convergence by testing the equality of the means of the first and last part of a Markov chain, was <5% for the three chains, which attests the convergence (Geweke, 1992).

Values of δ^13^C were multiplied by −1 before the statistical analyses. The normality of the data was assessed with the Shapiro–Wilk test, δ^15^N values were normal, while the δ^13^C values had to be transformed by the Box–Cox transformation to achieve normality. The δ^13^C and δ^15^N average values of the dry and wet dog foods were compared using the generalized linear model with a significance level of 1%.

## Results

**Figure 1:**
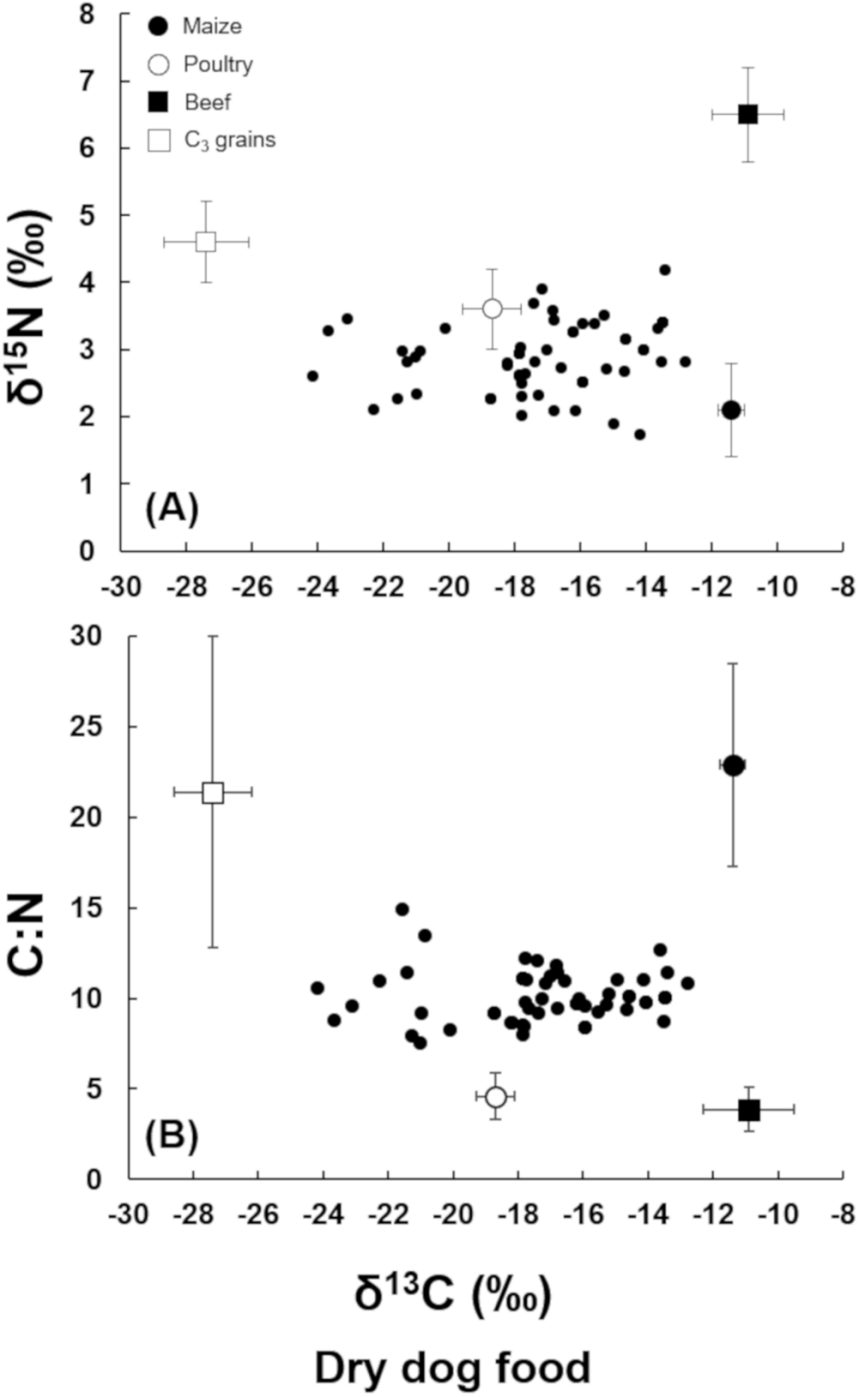
(A) Plot of δ^15^N vs. δ^13^C of the dry dog food samples. (B) Plot of C:N ratio vs. δ^13^C of the dry dog food samples. Dry dog food samples (multiple closed circles), average for C_3_ grains (open square, bars indicate standard deviation), average for poultry (open circle, bars indicate standard deviation), average for maize (closed circle, bars indicate standard deviation), average for beef (closed square, bars indicate standard deviation).

The δ^13^C values of dry dog food ranged from −24.2‰ to −12.8‰, with an average (± standard-deviation) of −17.1‰ ± 2.8‰ (Fig. 1). The δ^13^C values of wet dog food varied from −25.4‰ to −16.9‰, with an average (± standard-deviation) of −21.2‰ ± 2.4‰, which was significantly lower (*p* < 0.01) than the dog dry food. The δ^15^N values of the dry and wet food varied from 1.7‰ to 4.2‰, and from 0.5‰ to 5.5‰, respectively. The average δ^15^N values of dry food (2.9‰ ± 0.5‰) was not higher than the wet food (2.6‰ ± 1.3‰) (*p* > 0.01).

The plot of δ^15^N vs. δ^13^C shows that most of the dry dog food fell in an *iso-space* constrained by beef, grains, maize, and poultry, but with a clear trend to be clustered between poultry and maize ingredients (Fig. 1A). The plot of δ^13^C vs. C:N of dry dog food showed a similar trend; however, it seems that in this plot, bovine products could also be an important ingredient (Fig. 1B).

**Figure 2:**
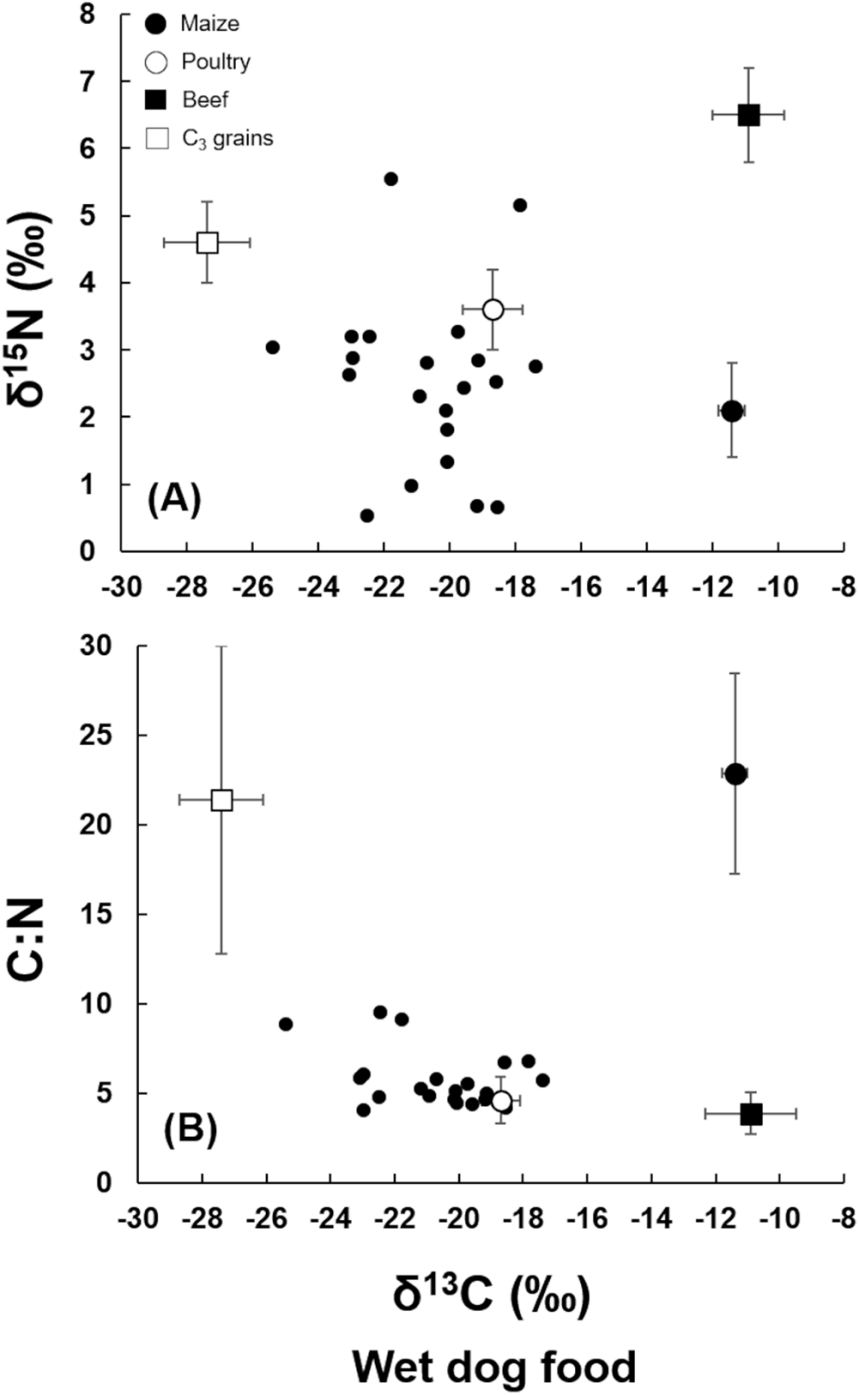
(A) Plot of δ^15^N vs. δ^13^C of the wet dog food samples. (B) Plot of C:N ratio vs. δ^13^C of the wet dog food samples. Wet dog food samples (multiple closed circles), average for C_3_ grains (open square, bars indicate standard deviation), average for poultry (open circle, bars indicate standard deviation), average for maize (closed circle, bars indicate standard deviation), average for beef (closed square, bars indicate standard deviation).

The same type of plot for the wet pet food showed that they contain higher proportions of grains compared with the dry food (Fig. 2A), as in general, δ^13^C values were lower (more negative). However, poultry still seemed to be the most important ingredient (Figs. 2A and 2B).

**Figure 3:**
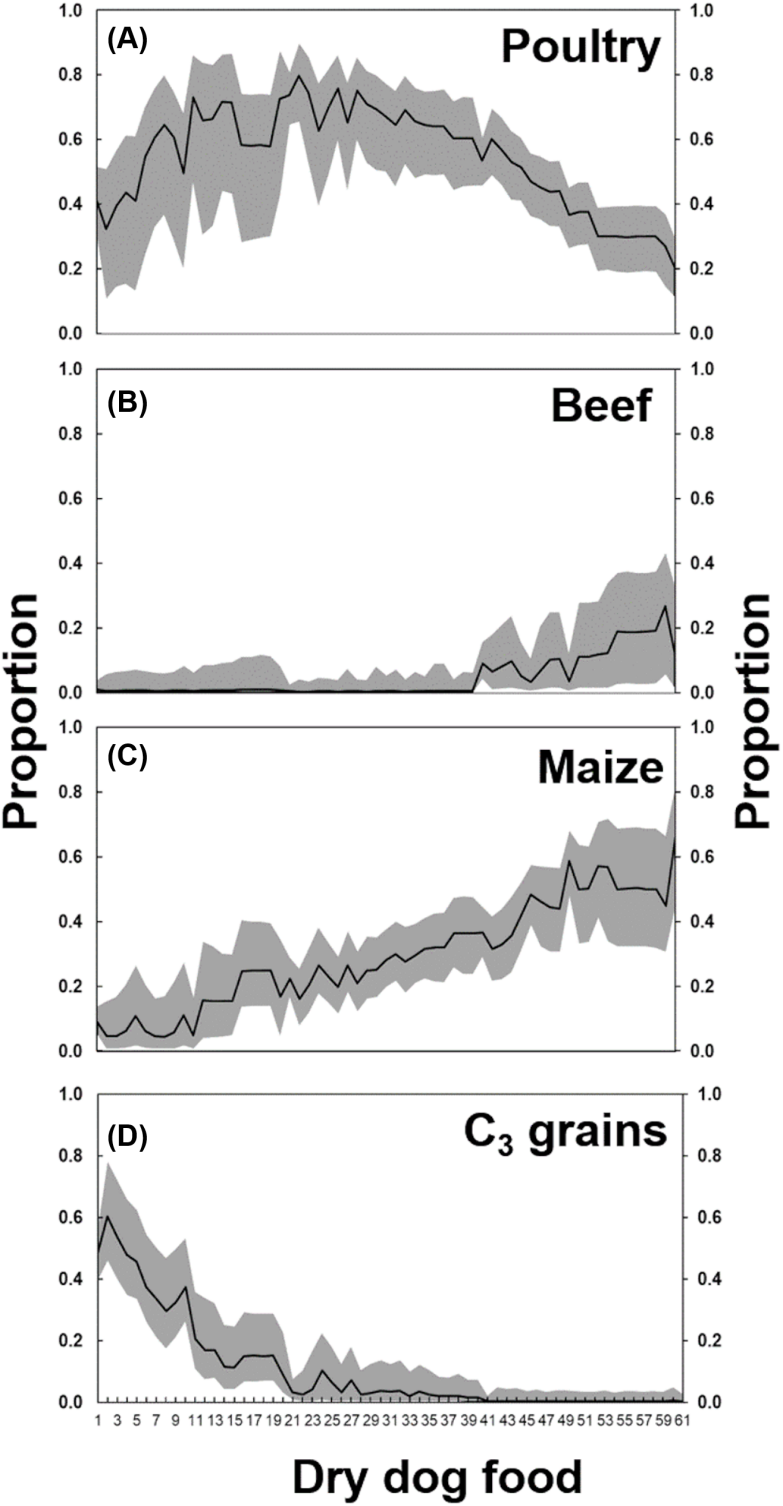
Proportion of (A) poultry, (B) beef, (C) maize, and (D) C_3_ grains, according to the MixSIAR analysis, in the dry dog foods produced in Brazil. The number in the *x*-axis refers to the “id” of dog foods listed in the Table 1. The solid line represents the median value, and the gray shaded area below the line the 5%-quantile and above the line the 95%-quantile.

The output of the MixSIAR clearly showed the low proportion of bovine products in dry dog food samples (Fig. 3). Based on the median value, the beef contribution was less than 0.05 in 70% of the samples. In 20% of the samples, the contribution was from 0.05 to 0.15 and in only 10% of them the contribution was higher than 0.15, with a maximum contribution of 0.27 in one of the samples (Table 1). On the other hand, poultry was the dominant ingredient in most of the samples (Fig. 3). The minimum contribution of poultry was 0.20 and the maximum reached 0.80. In more than 60% of the samples, the poultry contribution was 0.50 (Table 1). Maize was the second most important ingredient after poultry (Fig. 3), varying from a minimum of 0.04 to a maximum of 0.60. In almost 70% of the samples, maize contribution was higher than 0.20, and in 30% more than 0.40 (Table 1). Finally, C_3_ grains constituted a small proportion of dog food, however, still higher than bovine products (Fig. 3). In almost 70% of the samples, the contribution of C_3_ grains was lower than 10%. In only ∼20% of the samples, this contribution was higher than 30% (Table 1). The proportion of the ingredients with animal and plant origin in dog food was calculated by the summation of beef and poultry for animals and maize and grains for plants. The median of animal contribution to dry dog food was 0.60 (first quartile = 0.50; third quartile = 0.67), while the median of plant contribution was 0.40 (first quartile = 0.33; third quartile = 0.50).

**Figure 4:**
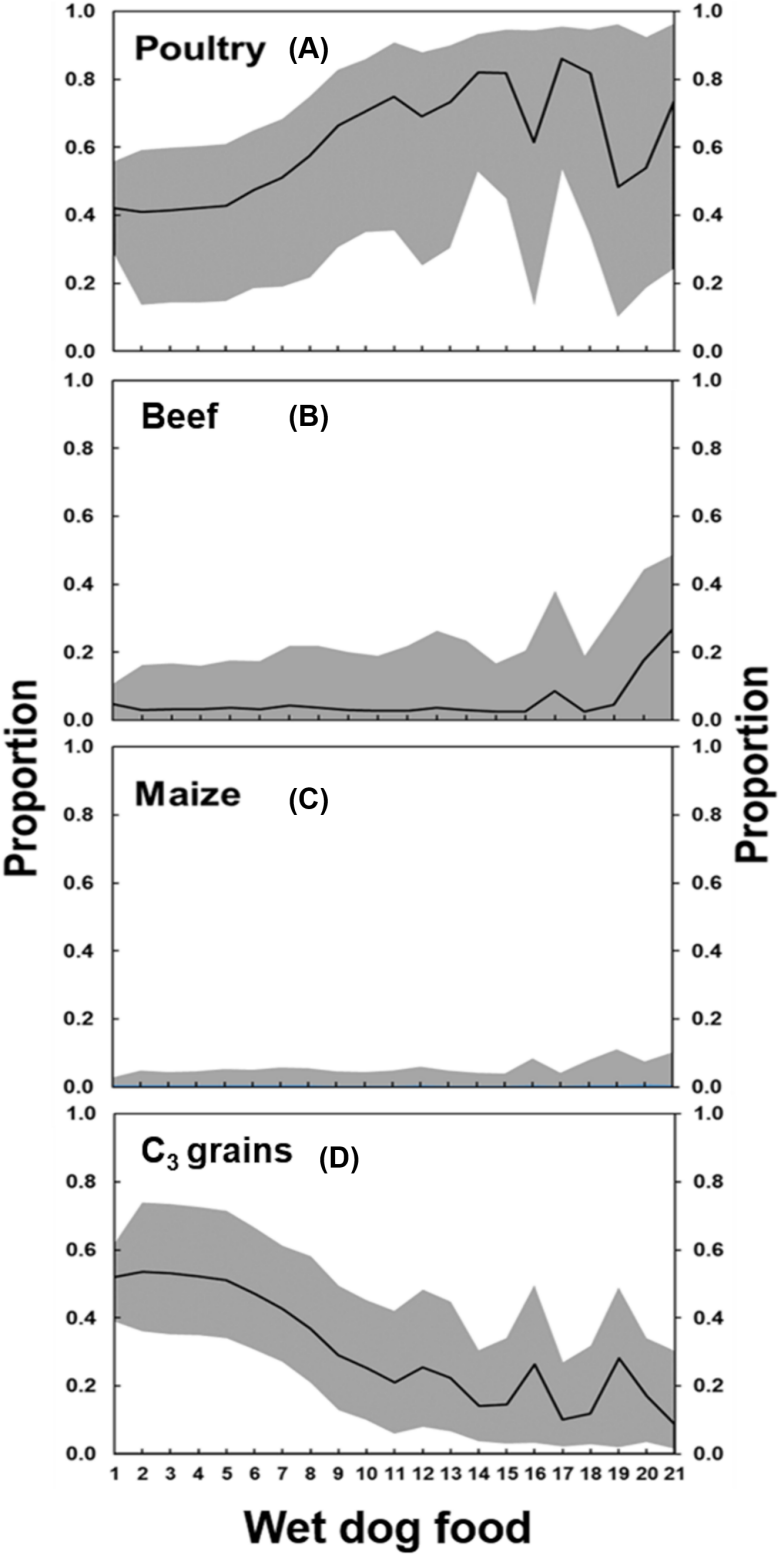
Proportion of (A) poultry, (B) beef, (C) maize, and (D) C_3_ grains, according to the MixSIAR analysis, in the wet dog foods produced in Brazil. The number in the *x*-axis refers to the “id” of dog foods listed in the Table 2. The solid line represents the median value, and the gray shaded area below the line the 5%-quantile and above the line the 95%-quantile.

The output of the MixSIAR model of the wet dog food showed a similar pattern to dry dog food (Table 2). The major difference was the much lower contribution of maize in the wet dog food compared to the dry dog food which was compensated by the higher proportion of C_3_ grains (Fig. 4). Animal vs. plant contribution in the wet dog food was similar to the dry dog food, with the median of animal contribution of 0.58 (first quartile = 0.42, third quartile = 0.73), and median of plant contribution of 0.42 (first quartile = 0.27, third quartile = 0.58).

## Discussion

It has been shown using stable isotope analysis that approximately 60% (ranging from 32% to 86%) of dog food content available in Brazil has an animal origin and 40% (ranging from 14% to 67%) has a vegetal origin, which is in line with other studies that reached the same conclusion but using other techniques (Murray et al., 1999; Weber, Biourge & Nguyen, 2017). This finding has several implications. First, it leads to the ongoing debate about eating habits of dogs. For obvious reasons, the dog food industry claims that dogs are omnivores (Hill’s Pet Nutrition, 2018), while concerned consumers claim that dogs are carnivores (Dog Food Advisor, 2018). There is no doubt that dogs were domesticated from wolves (Savolainen et al., 2002; Lindblad-Toh et al., 2005). Wolves are carnivores, and dogs, as their descendants, have inherited several aspects of wolf anatomy that identify them as carnivores. However, recent findings have shown that early ancestors of modern dogs acquired the ability to digest starch to cope with the advent of agriculture by humans (Axelsson et al., 2013; Bazolli et al., 2015; Weber, Biourge & Nguyen, 2017). Therefore, dogs are carnivores capable of digesting plants, and although it is accepted that diet can affect the incidence of diseases in dogs (Valenzuela et al., 2013), the long-term effects of eating food with high proportions of plants are unknown.

Among animal ingredients, poultry was by far the largest component of dog food produced in Brazil. Most labels do include poultry as an ingredient, not as meat but as meal or by-product, even in packages showing a picture of a savory piece of chicken. The fact that most of the dog food in Brazil is made of by-products is beneficial, avoiding direct competition between humans and dogs for food resources (Okin, 2017). However, like in the US, there is a trend in Brazil toward marketing premium dog food, which is part of the pet humanization process (Swanson et al., 2013). According to Kumcu & Woolverton (2010), this type of food is not only designed considering dog health, but also to link dogs to human attributes, like weight management, and the use of human grade ingredients. If this trend persists, depending on the quality of the ingredients, the competition between humans and dogs for food resources will intensify (Okin, 2017).

In this sense, the heavy use of plant products like maize meal by the pet food industry positions dogs in frank competition with humans for food. This is especially an important issue in countries like Brazil, where a significant proportion of the population is still undernourished (Martinelli et al., 2010). On the other hand, a high proportion of plant products can be beneficial for the environment, since it may be argued that the plant-based food chain is more efficient and has a lower environmental impact than the animal-based food chain (Pimentel & Pimentel, 2003), as there are higher greenhouse gases emissions during the production of animal products, compared with plant-based products, with even higher emissions associated with meats from ruminants (Carlsson-Kanyama & González, 2009).

Finally, an important finding of this study is the negligible presence of beef, meal or by-products in dog food made in Brazil. Most of the dog food claiming to have meat, either by means of a picture of a nice chunk of meat or by the inscription in the label “beef flavored,” had on average less than 20% of this ingredient in their composition (Fig. 3). The law in Brazil allows, in this case, producers to just add beef flavoring compounds, and not bovine products themselves. This fact clearly misleads consumers who generally buy dog food according to their standards, expecting beef in the food (Kumcu & Woolverton, 2010).

## Conclusion

Ingredients with animal and plant origin constitute approximately 60% (ranging from 32% to 86%) and 40% (ranging from 14% to 67%) of dog food in Brazil, respectively. Poultry and maize are the main ingredients. Poultry is added as by-products or meal, avoiding the competition for food between dogs and humans, while they can compete for maize. On the other hand, the large proportion of ingredients with plant origin decreases the energy and environmental footprint of the dog food, since they are less harmful compared to the ingredients with animal origin. Labels can mislead consumers by showing pictures of items that are not necessarily part of the product composition and by not showing the detailed information on the proportion of each ingredient. This would allow customers to make their own choices weighing their pet’s nutritional needs, the competition between animals and humans and environmental sustainability.

## Supplemental Information

10.7717/peerj.5828/supp-1 Supplemental Information 1 C_3_ grains and maize. C_3_ grains and maize isotopic and C:N data.

## Additional Information and Declarations

### Data Availability

The following information was supplied regarding data availability: The raw data are available in Tables 1 and 2.
